# Autophagy and apoptosis are regulated by stress on Bcl2 by AMBRA1 in the endoplasmic reticulum and mitochondria

**DOI:** 10.1186/s12976-019-0113-5

**Published:** 2019-10-29

**Authors:** Bojie Yang, Quansheng Liu, Yuanhong Bi

**Affiliations:** 1 School of Mathematical Sciences, Inner Mongolia University, Hohhot, 010021 China; 2 School of Statistics and Mathematics, Inner Mongolia, University of Finance and Economics, Hohhot, 010070 China; 3 Inner Mongolia Key Laboratory of Economic Data Analysis and Mining, Hohhot, 010070 China

**Keywords:** Autophagy, Apoptosis, B-cell lymphoma-2, AMBRA1, Endoplasmic reticulum, Mitochondria

## Abstract

**Background:**

Autophagy and apoptosis are two important physiological processes that determine cell survival or death in response to different stress signals. The regulatory mechanisms of these two processes share B-cell lymphoma-2 family proteins and AMBRA1, which are present in both the endoplasmic reticulum and mitochondria. B-cell lymphoma-2 family proteins sense different stresses and interact with AMBRA1 to regulate autophagy and apoptosis, which are respectively mediated by Beclin1 and Caspases. Therefore, we investigated how different levels of stress on B-cell lymphoma-2 family proteins that bind to AMBRA1 in the endoplasmic reticulum and mitochondria regulate the switch from autophagy to apoptosis.

**Methods:**

In this paper, we considered the responses of B-cell lymphoma-2 family proteins, which bind to AMBRA1 in both the endoplasmic reticulum and mitochondria, to two different levels of stress in a model originally proposed by Kapuy et al. We investigated how these two stress levels affect the transition from autophagy to apoptosis and their effects on apoptosis activation over time. Additionally, we analyzed how the feedback regulation in this model affects the bifurcation diagrams of two levels of stress and cell fate decisions between autophagy and apoptosis.

**Results:**

Autophagy is activated for minor stress in mitochondria regardless of endoplasmic reticulum stress, while apoptosis is activated for only significant stress in mitochondria. Apoptosis is only sensitive to mitochondria stress. The time duration before apoptosis activation is longer in the presence of high AMBRA1 levels with high endoplasmic reticulum and mitochondria stress. AMBRA1 can compete with B-cell lymphoma-2 family proteins to bind and activate Beclin1 and thus promote the autophagy process for a long time before apoptosis. Furthermore, apoptosis is prone to occur with increasing activation of Caspases, inactivation of Beclin1-A and the Michaelis constant of Caspases.

**Conclusion:**

A novel mathematical model has been developed to understand the complex regulatory mechanisms of autophagy and apoptosis. Our model may be applied to further autophagy-apoptosis dynamic modeling experiments and simulations.

## Background

Autophagy and apoptosis play crucial roles in deciding cellular survival and death in response to different stress signals, such as nutrient starvation and endoplasmic reticulum (ER) stress [1–3]. Autophagy, a cellular survival process, provides energy through degrading abnormal cytoplasmic components in the lysosomal pathway and can be activated by the Beclin1 protein in the ER [4–9]. However, excessive levels of autophagy can lead to apoptosis with increased stress levels [7, 10–13]. Apoptosis, a kind of programmed cell death, can be triggered by the proapoptotic protein Bax, which causes mitochondrial membrane permeabilization to release mitochondrial cytochrome c into the cytoplasm, further activating Caspases to induce apoptosis [14–17]. An increasing number of studies confirm that the autophagy and apoptosis networks are linked at various levels through common regulatory elements [18, 19].

B-cell lymphoma-2 (Bcl2) proteins in the mitochondria and ER are important regulators of autophagy and apoptosis [20–23]. Bcl2 in the ER (ER-Bcl2) and mitochondrial Bcl2 (mito-Bcl2) play different roles in activating the different responses of autophagy and apoptosis [24]. ER-Bcl2 negatively regulates the Beclin1-dependent autophagy program, while mito-Bcl2 has been shown to exert an antiapoptotic effect [25–27]. Several mathematical models of the autophagy-apoptosis network, including crosstalk between Bcl2 proteins, have been established [28, 29] to explore cell fate decisions [30–32]. Kapuy et al. presented a minimal model that contains interplay between crucial autophagy and apoptosis proteins; however, they did not compare simulations and experimental measurements. Tavassoly et al. addressed this disadvantage; however, the important AMBRA1 protein was not included in their assessment. The AMBRA1 protein translocates from mitochondria to the ER and regulates both Beclin1-dependent autophagy and apoptosis [33, 34]. AMBRA1 positively regulates the Beclin1-dependent autophagy program through functioning with ER-Bcl2 and mito-Bcl2 [35]. AMBRA1 binds preferentially to mito-Bcl2 under normal conditions; after autophagy initiation upon stress, AMBRA1 is released from mito-Bcl2 to ER-Bcl2, and binding to Beclin1 is increased to promote autophagy in the ER [36]. Therefore, AMBRA1, ER-Bcl2 and mito-Bcl2 should be included in the autophagy and apoptosis network in further analyses of cell fate decisions upon different stress levels.

Various stress signals in the ER and mitochondria can activate autophagy or apoptosis. For example, nutrient-induced stress of the ER activates autophagy to recycle damaged organelles [37, 38]. DNA damage can cause apoptosis through promoting the release of cytochrome c from the mitochondria to the cytosol [39–41]. Bcl2, a coregulator of autophagy and apoptosis in both the ER and mitochondria, can sense different stresses [42–44]. Therefore, it is important to explore how different levels of stress on ER-Bcl2 and mito-Bcl2 regulate the switch from autophagy to apoptosis. In this work, ER-Bcl2 and mito-Bcl2 protein binding to AMBRA1 is considered in a previously reported model [29], and different levels of stress are imposed in this model. Then, we focus on the effects of these two levels of stress on the switch from autophagy to apoptosis. The results are organized as follows. First, a new model is proposed. Second, typical time series of both active Beclin1 (Beclin-A) and Caspases and Caspases activation times are given for different stress levels, bifurcation analyses of these two stress levels are described, and the effect of feedback regulation intensity in this model on bifurcation diagrams is studied. Finally, we discuss the results and provide a conclusion.

## Methods

### Mathematical model

**Fig. 1:**
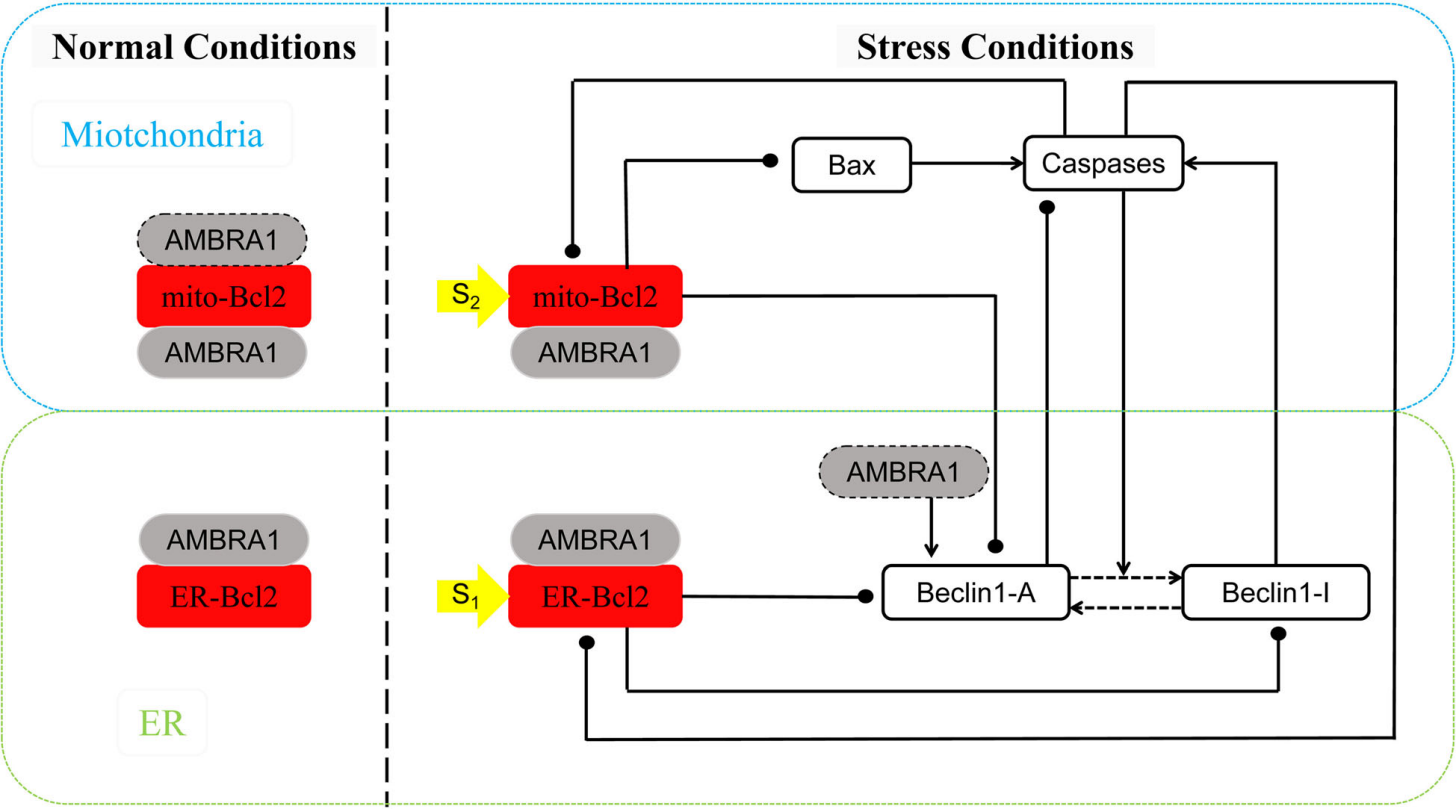
The autophagy-apoptosis network with two modules: the ER and mitochondria. The ER and mitochondria are represented by boxes outlined with green and blue dashed lines, respectively. State transitions are indicated by dotted lines with arrowheads, and promotion and inhibition are denoted by solid lines with arrowheads and dots, respectively

Here, we consider two different levels of stress (such as transient nutrient starvation, DNA damage or growth factor withdrawal) denoted by *S*_1_ and *S*_2_, respectively, on two modules: the ER and mitochondria, as shown in the network diagram in Fig. 1. Under normal conditions, AMBRA1 binds preferentially to mito-Bcl2 over ER-Bcl2. However, after autophagy induction under stress conditions, some AMBRA1 proteins disassociate from mito-Bcl2 and translocate into the ER to promote Beclin1-A activity. Notably, AMBRA1 binds to Bcl2 and promotes the degradation of Bcl2, but its Bcl2-binding rates in mitochondria and the ER are different. Additionally, Beclin1-A activity is promoted by AMBRA1. In the ER, Beclin1-A, an inducer of autophagy, can cotransform with an inactive form of Beclin1 (Beclin1-I). Beclin1-A and Beclin1-I deactivate and activate apoptosis-inducing Caspases, respectively, which in turn promotes the production of Beclin1-I. Therefore, there is a positive feedback loop between Caspases and Beclin1-I but a double-negative feedback loop between Caspases and Beclin1-A. In the mitochondria, Bax promotes the release of cytochrome c to activate Caspases. Caspases inhibit ER-Bcl2 and mito-Bcl2. Two stresses *S*_1_ and *S*_2_, are imposed on ER-Bcl2 and mito-Bcl2; while the former inhibits both Beclin1-A and Beclin1-I, the latter inhibits Beclin1-A and Bax.

### Dynamic equations

Based on their biochemical interactions shown in Fig. 1, the following eight components are considered: ER-BCL2 ([*Bcl*2_*e*_]), mito-BCL2 ([*Bcl*2_*m*_]), AMBRA1 ([AMBRA1]), Caspases ([Casp]), active Beclin1 ([Beca]), inactive Beclin1 ([Beci]), Bcl2_e_-Beclin1 complex ([Becac]) and Bcl2_m_-Bax complex ([Baxc]). The rate of every component is described by an ordinary differential equation (ODE) composed of production and consumption terms. The production term is a protein synthesis or activation term, while the consumption term is a protein degradation or inaction term. Every term on the right-hand side of the ODE corresponds to each biochemical reaction, which is described by using either the law of mass action or Michaelis-Menten kinetics, and the Michaelis constant Jcp is the substrate concentration at which the rate is equal to half of the maximal rate [45]. The unit of time is h, while protein concentrations are in arbitrary units. The significance and parameter values are shown in Table 1. In this work, the time series and bifurcation curves were computed numerically by XPP-AUT. The rate of every component is described by Eqs. (1)–(10) as follows:

1 $$ \frac{d\left[ Bcl{2}_e\right]}{dt}={k}_1-\left({k}_2+{k}_3\cdot {S}_1+{k}_4\cdot Casp+{k}_5\cdot AMBRA1\right)\cdot Bcl{2}_e $$

2 $$ \frac{d\left[ Bcl{2}_m\right]}{dt}={k}_1-\left({k}_2+{k}_3\cdot {S}_2+{k}_4\cdot Casp+{k}_6\cdot AMBRA1\right)\cdot Bcl{2}_m $$

3 $$ \frac{d\left[ AMBRA1\right]}{dt}={k}_7-\left({k}_8\cdot Bcl{2}_e+{k}_9\cdot Bcl{2}_m+{k}_{10}\right)\cdot AMBRA1 $$

4 $$ {\displaystyle \begin{array}{c}\frac{d\left[ Casp\right]}{dt}=\left({k}_{12}+{k}_{13}\cdot Beci+{k}_{14}\cdot \left( Baxt- Baxc\right)\right)\\ {}\cdot \left( Caspt- Casp\right)/\left( Jcp+ Casp t- Casp\right)\\ {}-\left({k}_{15}+{k}_{16}\cdot Beca\right)\cdot Casp/\left( Jcp+ Casp\right)\end{array}} $$

5 $$ {\displaystyle \begin{array}{c}\frac{d\left[ Beca\right]}{dt}={k}_{11}\cdot AMBRA1-{k}_a\cdot \left( Bcl{2}_e- Becac- Becic\right)\cdot Beca\\ {}-\left({k}_{18}+{k}_{19}\cdot Casp\right)\cdot Beca+\left({k}_b+{k}_2+{k}_3\cdot {S}_1+{k}_4\cdot Casp\right)\cdot Beca c\\ {}+{k}_{17}\cdot Beci\end{array}} $$

6 $$ {\displaystyle \begin{array}{c}\frac{d\left[ Beci\right]}{dt}=-{k}_a\cdot \left( Bcl{2}_e- Becac- Becic\right)\cdot Beci\\ {}+\left({k}_{18}+{k}_{19}\cdot Casp\right)\cdot Beca+\left({k}_b+{k}_2+{k}_3\cdot {S}_1+{k}_4\cdot Casp\right)\cdot Beci c\\ {}-{k}_{17}\cdot Beci\end{array}} $$

7 $$ {\displaystyle \begin{array}{c}\frac{d\left[ Becac\right]}{dt}={k}_a\cdot \left( Bcl{2}_e- Becac- Becic\right)\cdot Beca\\ {}-\left({k}_{18}+{k}_{19}\cdot Casp\right)\cdot Beca c-\left({k}_b+{k}_2+{k}_3\cdot {S}_1+{k}_4\cdot Casp\right)\cdot Beca c\\ {}+{k}_{17}\cdot Becic\end{array}} $$

8 $$ {\displaystyle \begin{array}{c}\frac{d\left[ Baxc\right]}{dt}={k}_c\cdot \left( Baxt- Baxc\right)\cdot \left( Bcl{2}_m- Baxc\right)\\ {}-\left({k}_d+{k}_2+{k}_3\cdot {S}_2+{k}_4\cdot Casp\right)\cdot Baxc\end{array}} $$

9 $$ Bcl2t= Bcl{2}_e+ Bcl{2}_m $$

10 $$ Becic= Bect- Beca- Beci- Beca c $$

**Table 1:** Parameters and their descriptions (protein concentrations are in arbitrary units, and the unit of time is h)

Parameter	Significance	Value
*k*_1_	rate of Bcl2 synthesis	0.05
*k*_2_	basal rate of Bcl2 degradation	0.01
*k*_3_	stress-dependent rate of Bcl2 degradation	0.6
*k*_4_	Caspases-dependent rate of Bcl2 degradation	0.1
*k*_5_	AMBRA1-dependent rate of ER-Bcl2 degradation	0.3
*k*_6_	AMBRA1-dependent rate of mito-Bcl2 degradation	0.4
*k*_7_	basal rate of AMBRA1 activation	0.001
*k*_8_	ER-Bcl2-dependent rate of AMBRA1 degradation	0.3
*k*_9_	mito-Bcl2-dependent rate of AMBRA1 degradation	0.4
*k*_10_	basal rate of AMBRA1 inactivation	0.01
*k*_11_	AMBRA1-dependent rate of Beclin1 activation	0.3
*k*_12_	basal rate of Caspases activation	0
*k*_13_	inactivated Beclin1-dependent Caspases activation constant	0.05
*k*_14_	Bax-dependent rate of Caspases activation	0.4
*k*_15_	basal Caspases inactivation constant	0.1
*k*_16_	Beclin1-dependent rate of Caspases inactivation	0.37
*k*_17_	Beclin1activation rate	1
*k*_18_	basal Beclin1inactivation rate	0.01
*k*_19_	Caspases-dependent rate of Beclin1 inactivation	5
Jcp	Caspases Michaelis constant	0.01
*k*_*a*_	rate of Bcl2-Beclin1 complex association	0.1
*k*_*b*_	rate of Bcl2-Beclin1 complex dissociation	1
*k*_*c*_	rate of Bcl2-Bax complex association	8
*k*_*d*_	rate of Bcl2-Bax complex dissociation	0.1
*S*_1_, *S*_2_	stress level *S*_1_ and *S*_2_	0.5
Bcl2t	total level of Bcl2	1
Baxt	total level of Bax	0.25
Caspt	total level of Caspases	1
Becic	inactive, Bcl2-bounded Beclin1	0.2
Bect	total level of Beclin1	1

## Results

We focused on exploring the effect of different stress levels on ER-Bcl2 and mito-Bcl2 on the transition between autophagy and apoptosis. First, typical time series and Caspases activation times are given for different stresses. Then, bifurcation analyses of the two stresses in this model are carried out under different feedback regulation conditions.

### Autophagy-apoptosis transition mediated by two different stresses on the ER and mitochondria

**Fig. 2:**
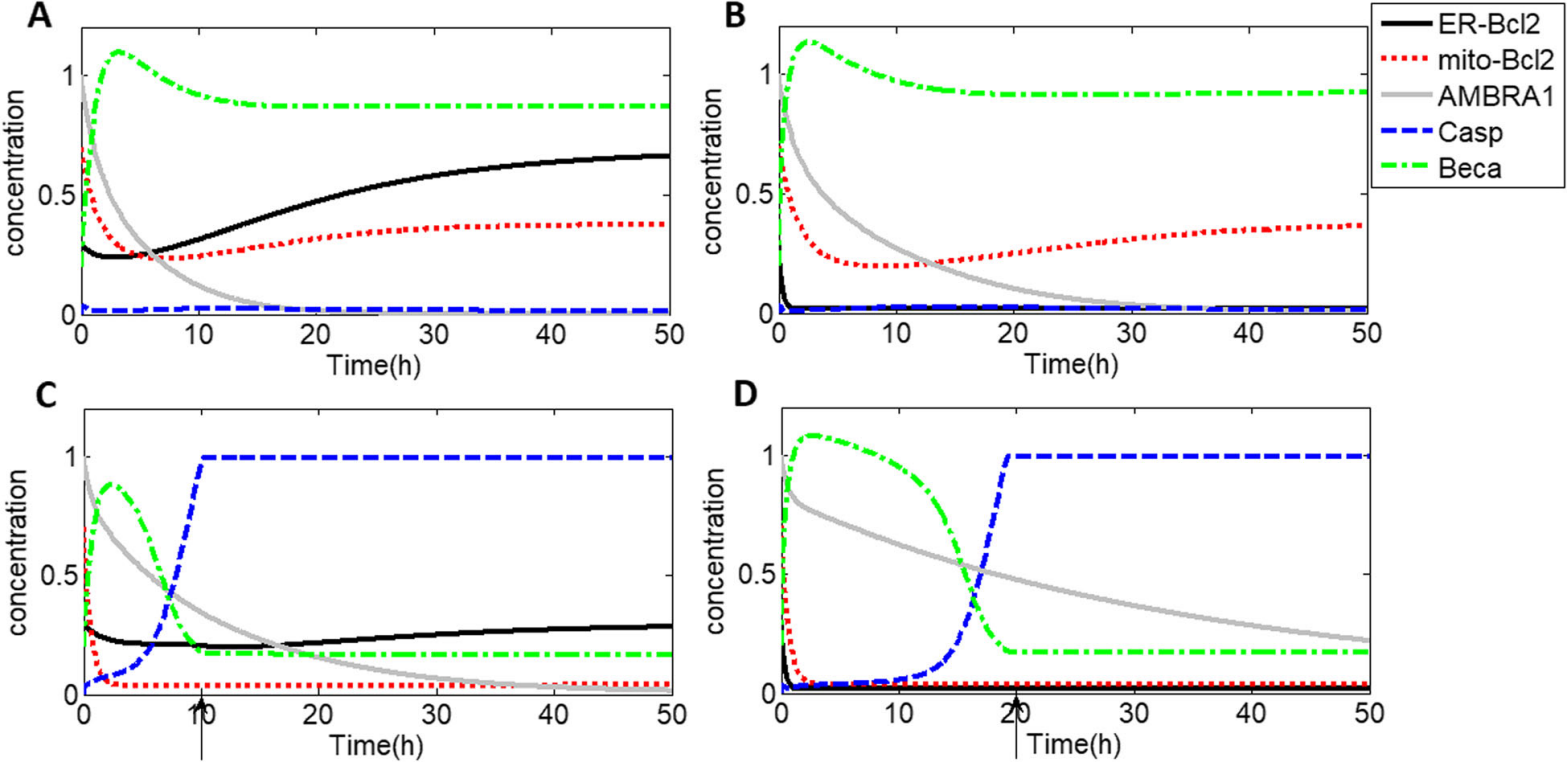
Time series of ER-Bcl2, mito-Bcl2, AMBRA1, Casp and Beca under different stress levels on the ER and mitochondria. **a** *S*_1_ = 0.1, *S*_2_ = 0.2; **b** *S*_1_ = 4.5, *S*_2_ = 0.2; **c** *S*_1_ = 0.1, *S*_2_ = 2; **d** *S*_1_ = 4.5, *S*_2_ = 2. The arrows indicate Casp activation times

In general, autophagy is activated first by Beclin1-A in the ER, and apoptosis is then activated by Caspases in the mitochondria, depending on both the intensity and duration of stress on the ER and mitochondria. In this section, we focus on how two stresses, *S*_1_ and S_2_, on ER-Bcl2 and mito-Bcl2, respectively, regulate the transition from autophagy to apoptosis. Without loss of generality, we explore the sensitive of autophagy or apoptosis to two stresses, *S*_1_ and S_2_, with different intensities, as shown in Fig. 2 by the time series of the concentrations of Bcl2_*e*_ (black, solid curve), Bcl2_*m*_ (short, red, dashed curve), AMBRA1 (gray, solid curve), Casp (long, blue, dashed curve) and Beca (green, dash-dot curve). First, at a low *S*_2_(*S*_2_ = 0.2), we consider low and a high value of *S*_1_(*S*_1_ = 0.1 and 4.5), as shown in Fig. 2a and b, respectively. An abrupt increase in Beca with either a low *S*_1_ or a high *S*_1_, which facilitates the degradation of ER-Bcl2 and dissociation of the Bcl2_e_-Beclin1 complex, can activate autophagy. Autophagy induction promotes the release of AMBRA1 from mito-Bcl2, which further promotes the Beclin1-A-dependent autophagy program. Furthermore, Beclin1-A and mito-Bcl2 deactivate Caspases to protect cells from death. Notably, although the high *S*_1_ value in Fig. 2b prompts ER-Bcl2 and mito-Bcl2 levels to first decrease rapidly and even remain very low, Caspases are still inactive due to the promotion of Beclin1-A activity in the ER by AMBRA1, promoting autophagy.

**Fig. 3:**
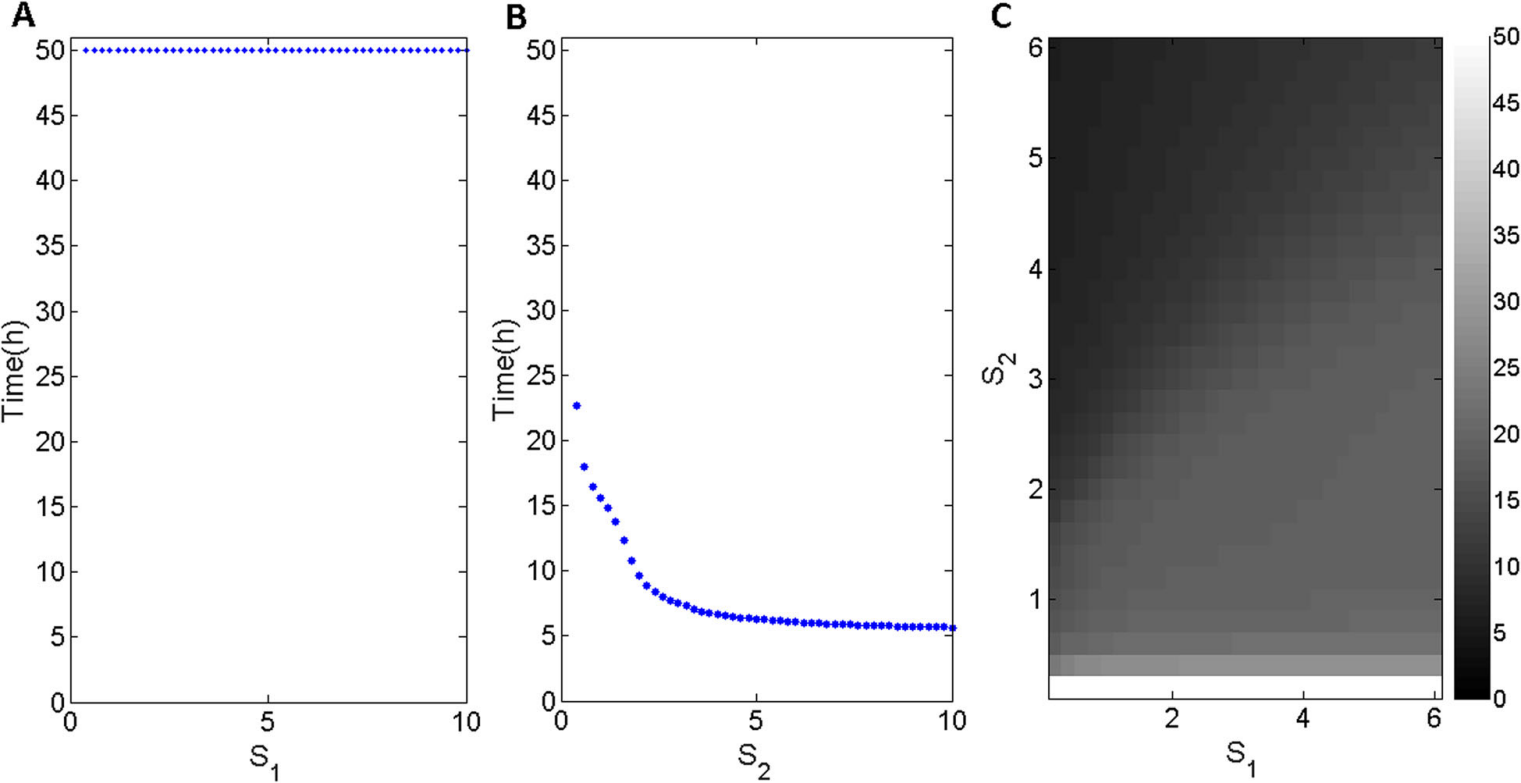
Time before apoptosis activation in which Casp reached its highest level for different values. **a** *S*_1_ at *S*_2_ = 0.2; **b** S_2_ at *S*_1_ = 0.1; **c** grayscale intensity showing the dependency of time on both *S*_1_ and *S*_2_

In contrast, at a high *S*_2_(*S*_2_ = 2), as shown in Fig. 2c and d, apoptosis with either a low (S_1_ = 0.1 in Fig. 2c) or high (*S*_1_ = 4.5 in Fig. 2d) value of S_1_ is activated very quickly by increasing Casp levels. In fact, autophagy is first activated by low levels of mito-Bcl2 and high levels of AMBRA1 and S_2_, while sustained levels of these molecules activate Caspases-induced apoptosis. Additionally, Casp reaches a high level (as labeled by arrows in Fig. 2) are 10 h and 20 h before apoptosis activation for a low S_1_ and a high S_1_, respectively. As shown in Fig. 2c and d, apoptosis can be activated at a higher S_2_, while the Caspases activation time is dependent on the value of S_1_, and the activation time is shorter for a low S_1_ (Fig. 2c) than for a high S_1_ (Fig. 2d). When there is a large difference between low stress levels in the ER and high stress levels in mitochondria, apoptosis can be easily activated. Otherwise, apoptosis activation is delayed with high stress levels on both the ER and mitochondria. This delay occurs because less inhibition of AMBRA1 due to low ER-Bcl2 and mito-Bcl2 levels maintains high AMBRA1 levels for a long time to active autophagy before apoptosis. Furthermore, we determine the dependence of time before apoptosis activation on the levels of two stresses, S_1_ and S_2_, as shown in Fig. 3.

At a low S_2_ = 0.2, as shown in Fig. 3a, apoptosis can never be activated with increasing S_1_. However, apoptosis will be activated when S_2_ is higher than 0.4 with a low S_1_ = 0.1, as shown in Fig. 3b. Additionally, the time before apoptosis activation shown in Fig. 3b decreases with increasing S_2_. All these results are displayed in the overall view of grayscale intensities of time as a function of both S_1_ and S_2_ in Fig. 3c. As shown in Fig. 3c, apoptosis can easily be activated in a short time with a high S_2_ and low S_1_.

As discussed above, low levels of stress on mitochondria activate only autophagy with any stress level on the ER, while high levels of stress on mitochondria can induce a transition from autophagy to apoptosis. Additionally, autophagy is first induced by a high Beca level accompanied by a low Casp level, and apoptosis is then activated when Casp gradually increases. Therefore, autophagy and apoptosis exhibit two steady states, and further necessitate analysis of the stability of these steady states through a bifurcation diagram of different stress levels is discussed in the next section.

### Autophagy and apoptosis are determined by bifurcations for different stresses on the ER and mitochondria

**Fig. 4:**
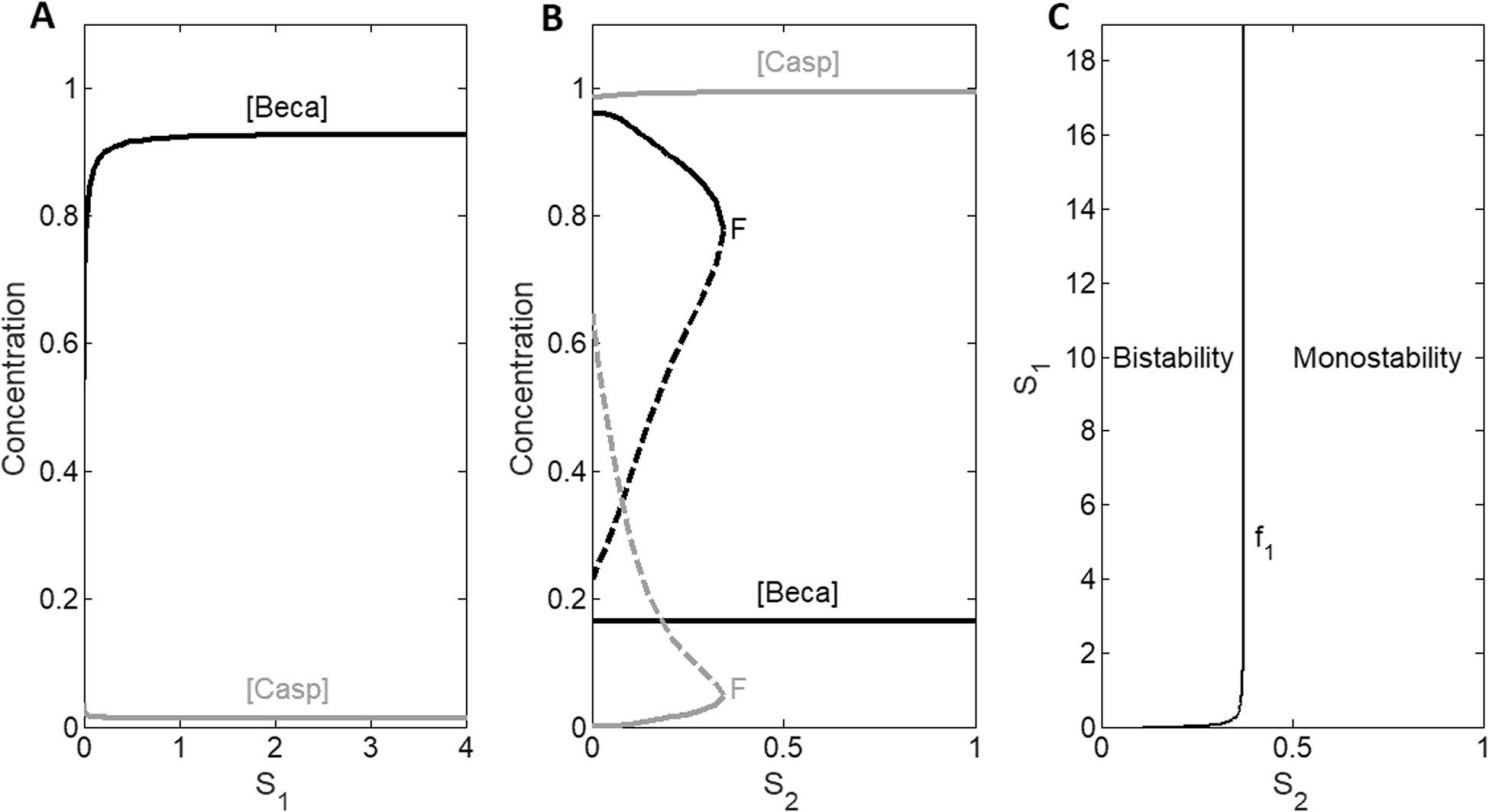
Bifurcation diagrams of both Beca (black lines) and Casp (gray lines). Codimension-one bifurcation curves with respect to **a** *S*_1_ at *S*_2_ = 0.2 and **b** *S*_2_ at *S*_1_ = 0.2. Stable steady states and unstable steady states are represented by solid and dotted lines, respectively. F is the equilibrium fold bifurcation point. **c** Codimension-two bifurcation diagram with respect to S_1_ and S_2_; the black line is the equilibrium fold bifurcation curve

Now, we show bifurcation diagrams of the steady state of Casp as well as Beca for two parameters, *S*_1_ and *S*_2_, in Fig. 4. As shown in Fig. 4a, codimension-one bifurcation curves of Beca and Casp with respect to the parameter S_1_ when S_2_ = 0.2 show only a stable steady state with a low Casp level and a high Beca level; this steady state corresponds to the autophagy process. However, the bifurcation curves of Beca and Casp with respect to the parameter S_2_ when S_1_ = 0.2 shown in Fig. 4b are bistable switch curves, in which upper and lower branches of the curves are composed of stable steady states separated by middle branches composed of unstable steady states. With a low value of S_2_, one of two stable steady states corresponds to a high Beca level but a low Casp level for autophagy, while the other corresponds to a low Beca level but a high Casp level for apoptosis. Furthermore, an increase in *S*_2_ leads to a transition from bistability to monostability via fold bifurcation point F, and Casp adopts a high steady state for apoptosis. Additionally, a high Casp steady state cannot return to a low steady state of autophagy with decreasing stresses because the other fold bifurcation point does not appear in the positive half-axis of the x-axis.

Furthermore, the codimension-two bifurcation diagram of *S*_1_ and *S*_2_ in Fig. 4c is divided into two regions by the fold bifurcation curve f_1_: monostability on the right region corresponds to apoptosis, and bistability on the left region denotes autophagy or apoptosis. Additionally, the f_1_ curve is an almost vertical line with increasing *S*_2_ and intersects the x-axis at only *S*_2_ = 0.16, which indicates that the system is sensitive to only *S*_2_. In addition, the codimension-two bifurcation diagram can be affected by feedback regulation, which is discussed in the following section.

### The effect of feedback regulation on autophagy and apoptosis

**Fig. 5:**
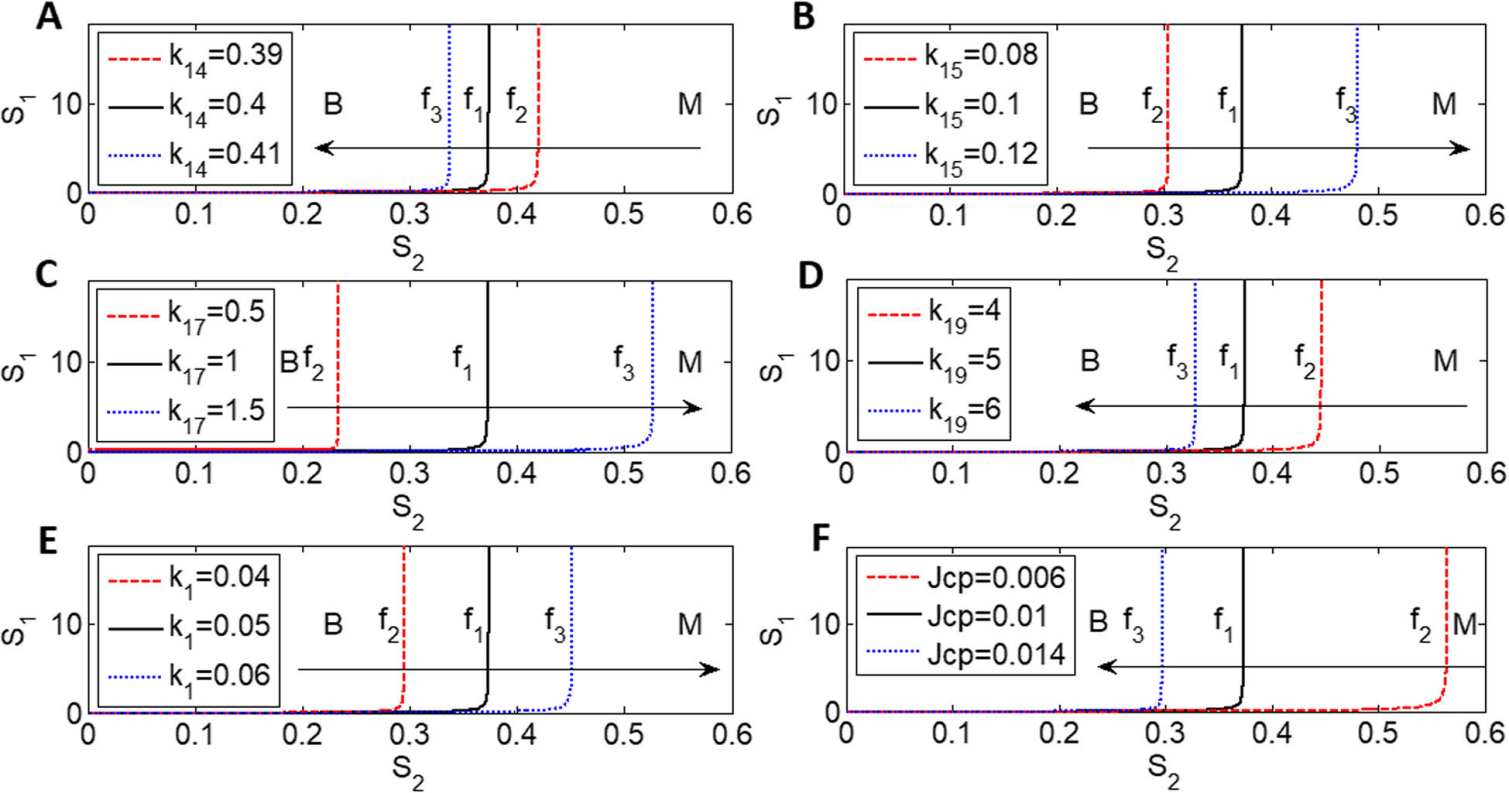
Codimension-two bifurcation diagrams of S_1_ and *S*_2_ for different levels of feedback regulation. **a** k_14_; **b** k_15_; **c** k_17_; **d** k_19_; **e** k_1_; **f** Jcp. B and M denote bistability and monostability, respectively

In this section, we explore the effect of all feedback regulation parameters on the codimension-two bifurcation diagrams of *S*_1_ and *S*_2_. All parameters are divided into three groups; the first two groups are related to the activation and inhibition of both Caspases and Beclin1-A, respectively (Fig. 5a–d), and the third group is associated with Bcl2 (Fig. 5e–f). Only one bifurcation diagram for each parameter is shown in every group due to similar effects. Among the Caspases activation rates *k*_12_, *k*_13_, *k*_14_, codimension-two bifurcation diagrams of *S*_1_ and *S*_2_ for different *k*_14_ values are shown in Fig. 5a; the fold bifurcation curves move left, and the monostable region is enlarged for an increased *k*_14_ activation rate. A higher activation rate increases the Casp level and then activates apoptosis with a low *S*_2_ value. In contrast, a high Caspases inactivation rate, *k*_15_, shifts the fold bifurcation curve right and reduces the monostable region on the bifurcation diagram in Fig. 5b, which is similar to the parameter *k*_16_. Therefore, apoptosis can be activated with only high *S*_2_ values when Caspases inactivation rates are high.

In contrast, based on Fig. 5c and d, the fold bifurcation curve moves right and left with an increase in the activation (*k*_17_) and degradation (*k*_18_, *k*_19_) rates of Beclin1-A, respectively. A high Beclin1-A activation rate increases the level of Beca to activate autophagy for the large bistable region, while a high Beclin1-A inactivation rate decreases the Beca level to easily activate apoptosis for the large monostable region. All parameters related to Bcl2 in the third group have little effect on the codimension-two bifurcation diagrams, except *k*_1_ and Jcp. A high *k*_1_ value moves the fold bifurcation curve to the right for autophagy, while a high Jcp value moves the fold bifurcation curve to the left for apoptosis.

## Discussion

Autophagy and apoptosis play essential roles in making cell fate decisions between life and death under stress. Autophagy promotes cell survival through activating Beclin1-A in the ER and can switch to apoptosis when Caspases in the mitochondria are activated. The Bcl2 and AMBRA1 proteins in both the ER and mitochondria act as important regulators of autophagy and apoptosis.

In this study, we added Bcl2 and AMBRA1 in the ER and mitochondria to an original model proposed by Kapuy et al. and investigated how two different stresses on Bcl2 in the ER (*S*_1_) and mitochondria (*S*_2_) affect the transition from autophagy to apoptosis. Based on typical time series and bifurcation analyses, we concluded that autophagy is activated upon a low level of stress on mitochondria regardless of t he level of stress on the ER (Fig. 2a, b), while apoptosis is activated for a high level of stress on the mitochondria (Fig. 2c, d). Normally, AMBRA1 partially localizes to the mitochondria and translocates to the ER to activate Beclin1 when autophagy is induced. In Fig. 2c, autophagy is maintained for a short time but then turns to apoptosis quickly because the AMBRA1 protein can compete with both ER-Bcl2 and mito-Bcl2 to bind and activate Beclin1. However, in Fig. 2d, autophagy is maintained for a long time before slowly switching to apoptosis. This occurs because higher AMBRA1 levels due to less inhibition by lower mito-Bcl2 and ER-Bcl2 levels play a positive role in maintaining autophagy for a much longer time (Fig. 2d). The delay in switching from autophagy to apoptosis shown in Fig. 2c and d was caused mainly by AMBRA1 under stress conditions. Also, the delay for different stresses can be seen clearly in Fig. 3c. Furthermore, apoptosis is sensitive to only S_2_, which is the key factor that divides the areas of the parameter plane (S_1_ and S_2_) into bistability and monostability (Fig. 4c). Apoptosis is prone to occur with an increased Caspases activation rate, Beclin1-A deactivation rate and Caspases Michaelis constant (Fig. 5). In summary, under two different stress levels on mito-Bcl2 and ER-Bcl2, the process of autophagy is promoted and maintained by AMBRA1 in the ER, while apoptosis is decided mainly by the stress on mitochondria.

Autophagy and apoptosis are important cellular responses to pharmacological interventions for diseases that are controlled by a dynamic network of interacting proteins. It is important to identify and target key components of this network when designing therapeutic regimens for diseases. In this work, Bcl2 and AMBRA1 in the ER and mitochondria are included in a previously described model, and we explored cellular responses to different levels of stress on the ER and mitochondria and feedback regulation in the network. However, the inclusion of more proteins in a more complete autophagy-apoptosis network is necessary, and cell fate in response to different conditions should be quantitatively analyzed.

## Conclusion

A mathematical model of autophagy and apoptosis regulated by stress on the binding of Bcl2 with AMBRA1 in the ER and mitochondria has been established. This model links experimental evidence and theoretical biology for a more comprehensive understanding of the complex regulatory mechanisms of autophagy and apoptosis. Therefore, our work may provide an application for further experiments and simulations of dynamic autophagy-apoptosis models.

## Data Availability

All data generated or analyzed during this study are included in this published article.

## Abbreviations

Baxc: Bcl2_m_-Bax complex
Bcl2: B-cell lymphoma-2
Bcl2_*e*_: B-cell lymphoma-2 in the endoplasmic reticulum
Bcl2_*m*_: B-cell lymphoma-2 in the mitochondria
Beca: Active Beclin1
Becac: Bcl2_e_-Beclin1 complex
Beci: Inactive Beclin1
Beclin1-I: Inactive Beclin1
Beclin-A: Active Beclin1
Casp: Caspases
ER: Endoplasmic reticulum
ER-Bcl2: B-cell lymphoma-2 in the endoplasmic reticulum
mito-Bcl2: B-cell lymphoma-2 in the mitochondria
ODE: Ordinary differential equation
